# Protective effects of inhalation of essential oils from *Mentha piperita* leaf on tight junctions and inflammation in allergic rhinitis

**DOI:** 10.3389/falgy.2022.1012183

**Published:** 2022-12-12

**Authors:** Nayoung Park, Jae Yoon Chung, Mi Hye Kim, Woong Mo Yang

**Affiliations:** Department of Convergence Korean Medical Science, College of Korean Medicine, Kyung Hee University, Seoul, South Korea

**Keywords:** allergic rhinitis, essential oils, *Mentha piperita* leaf, tight junction, inflammation, network pharmacology

## Abstract

Allergic rhinitis is one of the most common diseases, which is caused by IgE-mediated reactions to inhaled allergens. Essential oils from the *Mentha piperita* leaf (EOM) are known to be effective for various diseases, such as respiratory diseases. However, the effect of inhalation of EOM on tight junctions and inflammation related to allergic rhinitis is not yet known. The purpose of this research was to explain the effects of the inhalation of EOM on tight junctions and inflammation of allergic rhinitis through network pharmacology and an experimental study. For that purpose, a pharmacology network analysis was conducted comprising major components of EOM. Based on the network pharmacology prediction results, we evaluated the effect of EOM on histological changes in mice with ovalbumin and PM10-induced allergic rhinitis. Allergic symptoms, infiltration of inflammatory cells, and regulation of ZO-1 were investigated in mice with allergic rhinitis. Other allergic parameters were also analyzed by reverse transcription polymerase chain reaction and western blot in nasal epithelial cells. In the network analysis, the effects of EOM were closely related to tight junctions and inflammation in allergic rhinitis. Consistent with the results from the network analysis, EOM significantly decreased epithelial thickness, mast cell degranulation, goblet cell secretion, and the infiltration of inflammatory cells in nasal tissue. EOM also regulated the MAPK-NF-κB signaling pathway, which was related to tight junctions in nasal epithelial cells. This research confirmed that inhalation of EOM effectively restores tight junctions and suppresses inflammation in the allergic rhinitis model. These results reveal that EOM has a therapeutic mechanism to treat allergic rhinitis.

## Introduction

Allergic rhinitis is one of the most common diseases in the world (1). The symptoms of allergic rhinitis have been known to include rubbing, sneezing, rhinorrhea, nasal obstruction, and nasal itching, which are caused by IgE-mediated reactions to inhaled allergens. The prevalence of allergic rhinitis has increased to 11%–24% of the population, according to the World Health Organization and International Study of Asthma and Allergies in Childhood (2, 3).

Previous studies have found that environmental factors affect allergic diseases. In particular, exposure to air pollutants, one of the environmental factors, is associated with an increased risk of respiratory and allergic diseases (4). Ambient particulate matter (PM; particles with an aerodynamic diameter of <2.5 and 10 µm, PM10 and PM2.5) and nitrogen dioxide (NO_2_), which is a cause of air pollution, is known to be associated with allergic rhinitis (5). The components of PM trigger local and systemic inflammatory responses, which exacerbate the symptoms of allergic rhinitis (6). These negative effects of PM have been reported to attribute to nasal mucosal irritation, induce inflammation, cause cellular injury, and disrupt epithelial barrier function (7).

The initiation of allergic rhinitis is featured as disruption of the epithelial barriers through increases in the exposure of the nasal tissues to environmental allergens (8). In epithelial tissues, cell-cell interactions are mediated by the regulation of paracellular traffic, such as junctional complexes that consist of tight junctions for epithelial barrier function (9). Tight junctions, the most apically located of the paracellular junctional complexes, are composed of zonula occludins-1 (ZO-1), the claudins (CLDNs) family, occludin, and junctional adhesion molecules (JAMs), which connect transmembrane proteins to the cytoskeleton (10). These are important for all types of cellular processes, including the regulation of the epithelial tight junction assembly for functional permeability barrier (11).

The medicines for allergic rhinitis are the H1 receptor antihistamine, intranasal corticosteroid, intranasal antihistamine, and oral antihistamine, which are known to restore the epithelial barrier by acting on the tight junctions (12, 13). However, the long-term use of allergic rhinitis medication is known to induce several side effects, such as sleepiness, diarrhea, giddiness, and sedation (14). Recently, the inhalation of essential oils from natural plants by aerosol spray, nebulizing, and nasal treatment has been used as a treatment for respiratory diseases, because the inhalation of essential oils has been regarded as a health enhancer (15). Essential oils, which are known as insoluble plant extracts, have anti-inflammatory, antioxidant, and antimicrobial activities (16). Essential oils from the *Mentha piperita* leaf (EOM) contain phenol compounds and flavonoids that are known to be effective for various diseases, such as respiratory diseases, digestive diseases, disorders of the nervous system, and allergic disease (17). In our previous study, EOM recovered the airway inflammation response in asthma (18). This study was conducted to investigate the therapeutic efficacy of peppermint oil on the disruption of the tight junctions of the nasal epithelial barrier and airway inflammation in allergic rhinitis.

As a major role in network pharmacology, a network analysis based on widely existing databases enables the prediction of potential molecular mechanisms (19). A network pharmacology analysis can also be used to discover biomarkers, which play a crucial role in diseases (20). This study conducted a network pharmacological analysis to explore the potential compounds and target gene/protein/pathway mechanisms of an EOM-related tight junction recovery of the nasal epithelial barrier and airway inflammation in allergic rhinitis. Based on the results of the network pharmacology, the effects of EOM on tight junction disruption and inflammation in allergic rhinitis was confirmed via in vivo and in vitro experiments.

## Materials and methods

### Network construction of essential oil from *Mentha piperita* leaf and comparison of allergic rhinitis gene set

The EOM network was constructed with genes related to EOM constituents menthone, menthol, and methyl acetate (Pubchem CID: 1254, 26447, and 27867). The related genes were collected through PubChem (https://pubchem.ncbi.nlm.nih.gov/) chemical-gene co-occurrences in the literature (Supplementary Table S1). Eliminating the duplicates, a total of 155 genes were shown as chemical-gene co-occurrences with EOM. The allergic rhinitis-related genes were extracted using GeneCards (http://www.genecards.org/) to create the allergic rhinitis gene set. Using “allergic rhinitis” as a keyword in the GeneCards database, a total of 2,087 genes comprised the allergic rhinitis gene set. Each gene of the EOM network and allergic rhinitis gene set was counted, and overlapping genes were sorted.

### Functional enrichment analysis

Biological processes associated with the targets of the EOM network were investigated using Cytoscape (version 3.9.0). The pathways related to the EOM network were categorized by the Kyoto Encyclopedia of Genes and Genomes (KEGG) pathway 2021 human and Gene Ontology (GO) Process and were collected. Allergic rhinitis-related biological pathways were selected and organized, and the number of matched genes was counted.

### Preparation of essential oil from *Mentha piperita* leaf extraction for experiments

*Menta piperita* leaves were purchased from Dong-Yang Herb, Inc. (Seoul, Korea). The essential oil was extracted using the hydro-distillation method. 100 g of *Menta piperita* was filled in 1 L round-bottom flask with a Clevenger apparatus. Steam distillation was performed at 100 °C for 6 h, and 1.6 ml of the volatile oil was obtained (yield of EOM: 1.60%). The EOM was preserved in a vial at 4 °C for further use. A voucher specimen (01-02-01-KR-191217) was deposited at the Department of Convergence Korean Medical Science, College of Korean Medicine, Kyung Hee University, Seoul, Republic of Korea.

### Identification of constituents from essential oil from *Mentha piperita* leaf extraction

The EOM components of the extracted volatile oil from the *Menta piperita* leaf were determined using gas chromatography/mass spectrometry (GC-MS) analysis. The GC-MS analysis of essential oils was performed using the GC-2010 Shimadzu system. The two devices, the GC-2010 Shimadzu system and GCMS-QP2010 Plus quadrupole mass spectrometer (Agilent Technologies Inc., Santa Clara, CA, USA), were combined and analyzed. Samples of 2 µl (40 mg of oil dissolved in 1.5 ml of dichloromethane) were injected in a split mode at a ratio of 5:1. The oil components were separated through a 30-m HP-5MS capillary column (with a diameter of 0.25 mm and thick stationary phase film of 0.25 μm). The method of analysis was a programmed injection syringe into the headspace, and a volatile sample was injected into the GC. The column oven was kept at an initial temperature of 45 °C (3 min). The injector temperature was 250 °C and held for 20 min. The oil was scanned in the range of 100–500 *m*/*z*, and menthone, menthol, and methyl acetate were detected with peaks of 1, 3, and 5, respectively (Supplementary Table S1).

### Treatment of animals

The experimental procedures were performed by the Institutional Animal Ethics Committee of Kyung Hee University in Korea [KHUASP(SE)-20-363;2020-10-27]. Female BALB/c mice aged 5 weeks were purchased by DBL Co. (Eumseong, Korea). The mice were maintained with 12 h/12 h light/dark cycle (mean temperature 22 ± 2 °C, mean humidity 55% ± 10%). The BALB/c mice were randomly divided into five groups: (1) CTR: no-treatment control group; (2) OVA + PM10: ovalbumin (OVA) and PM10-treated group; (3) DEX: OVA and PM10-treated mice and inhaled with dexamethasone 0.06 (% w/v) as the positive control group; (4) EOM 0.0004: OVA and PM10-treated mice and inhaled with the EOM 0.0004 (% v/v) group; and (5) EOM 0.04: OVA and PM10-treated mice and inhaled with the EOM 0.04 (% v/v) group. The DEX and EOM groups inhaled a saline mixture using a self-made chamber with a nebulizer (Philips, Amsterdam, the Netherlands). The treatment was conducted three times per week for 5 min for 7 weeks. The treatment of DEX and EOM was pre-treated days 7–28. The DEX and EOM groups were then administered inhalation methods using a saline mixture on days 28–56. In order to induce the allergic rhinitis model, 10 mg of OVA (albumin in chicken eggs) and 500 mg of aluminum hydroxide (aluminum injection) were injected into the intraperitoneal injection with a total of 0.1 ml of saline solution on days 28, 35, and 42. Allergic rhinitis models were then challenged with 1 mg of OVA and 100 μg of PM10 in 50 μl of saline by intranasal instillation on days 49–51. At the end of the experiment, these models were sacrificed on day 56.

### Histopathological evaluation of the nasal cavity

The nasal tissue was removed, fixed in 10% neutralized formalin for 24 h, and then dehydrated using ethanol and xylene. The dehydrated nasal tissue was then cut into pieces with a thickness of 5 μm. The epithelial thickness and inflammatory cells were observed by staining nasal tissue with hematoxylin and eosin (H&E). Moreover, the nasal tissues were stained with periodic acid-Schiff (PAS) and toluidine blue for the detection of mucin in goblet cells and the degree of mast cell hyperplasia in the nasal section.

### Measurement of nasal symptoms

After the last OVA challenge, the number of times nasal rubbing and sneezing occurred was recorded by five observers blinded for 30 min to evaluate the allergic reaction. The actions of the mice were recorded by a video camera. The average values for the measurements made by the five observers were used for the statistical analysis.

### Evaluation of IgE and IgG2a by ELISA

Blood samples of the mice were obtained from the eyes immediately. The blood samples were collected with a centrifuge at 1,200 rpm for 30 min at 4 °C to separate the serum samples. The levels of IgE and IgG2a in the animals were evaluated by a commercial ELISA kit (BD Biosciences, San Diego, CA, USA), according to the manufacturer's recommendations.

### Cell counts for nasal lavage fluid

After the sacrifice, 1 ml of sterile saline solution was gently injected into the nasal cavity through the trachea and washed. The nasal lavage fluid (NALF) cells were collected through a centrifuge. The differential cells, such as macrophages, eosinophils, neutrophils, and lymphocytes, were stained with Wright-Giemsa and counted with a hemocytometer.

### Immunohistochemistry

For the immunohistochemistry (IHC) analysis, the nasal tissues were paraffine-sectioned into a thickness of 5 μm using a microtome. After being sectioned, the nasal tissues were incubated with 3% H2O2 for 30 min for endogenous peroxidase activities. The sections were blocked with a blocking solution for 1 h. The first antibody ZO-1 (1:500, Santa-Cruz, United States) was then added to the nasal tissues overnight at 4 °C. The sections were covered with a secondary antibody IgG rat (1:200, VECTOR Laboratories, USA) for 60 min at room temperature. They were stained with a DAB solution (Sigma-Aldrich, D5637) for immunoreactivity. Finally, the nuclei were stained with hematoxylin for counterstaining.

### Cell culture

RPMI 2650, a human nasal septum tumor epithelial cell line, was purchased from the Korean Cell Line Bank (Seoul, Korea). RPMI 2650 cells were grown in RPMI 1640 (Corning, USA) supplemented with 10% fetal bovine serum (FBS) and 1% penicillin/streptomycin in a CO_2_ atmosphere of 37% and 5%. The culture medium was changed every 2 days.

### Cytotoxicity of EOM

RPMI 2650 cells were seeded in 96-well plates at 3 × 10^4^ cells/well until a confluence of 80% was reached. The cells were treated with EOM using various concentrations of 10^−7^, 10^−6^, 10^−5^, and 10^−4^/ml (% v/v). RPMI 2650 cells were incubated for 24 h at 37 °C and in a CO_2_ atmosphere of 5%. After incubation, the cells were left with an MTT reagent (5 μg/ml final concentration) at 37 °C for 2 h. Formazan, known as a cell viability indicator, was dissolved through 100 ul of DMSO (Sigma Aldrich, Seoul, Korea) per well. The production of formazan was measured by an ELISA reader at 570 nm.

### RNA extraction and reverse transcription polymerase chain reaction

RPMI 2650 cells were exposed to 100 μg/ml of PM10 in the presence of 10^−7^, 10^−6^, and 10^−5^/ml (% v/v) of EOM for 24 h. Urban dust, SRM 1649b, was provided by the National Institute of Standards and Technology (Gaithersburg, MD, USA). RPMI 2650 cells were extracted in a TRIZOL reagent (Invitrogen, Carlsbad, CA, USA). The complementary DNA (cDNA) was synthesized by Maxime RT premix (Invitrogen Corp., Carlsbad, CA, USA). The synthesized cDNA was then evaluated by RT-PCR using a Maxime PCR premix kit (Invitrogen Corp.). The expression of PCR products was detected by 2% agarose gel and was stained with ethidium bromide. The relative band of target genes was determined by an Image J computerized densitometry system.

### Western blot analysis

RPMI 2650 cells were exposed to 100 μg/ml of PM10 in the presence of 10^−7^, 10^−6^, and 10^−5^/ml (% v/v) of EOM for 24 h. Urban dust, SRM 1649b was purchased by the National Institute of Standards and Technology (Gaithersburg, MD, USA). RPMI 2650 cells were extracted in a RIPA buffer (Thermo Scientific, Rockford, IL, USA). In total, 10 μg cell protein from RPMI 2650 cells were determined using the Bradford method. The protein was separated using sodium dodecyl sulfate–polyacrylamide gel electrophoresis and then transferred to the polyvinylidene difluoride (PVDF) membrane. The PVDF membranes were blocked by adding 3% bovine serum albumin to Tris-buffered saline (TBS) containing 0.1% of Tween-20 (TBS-T), and the primary antibody was incubated at 4 °C overnight. After binding to the PVDF membrane for 1 h using horseradish peroxidase-conjugated secondary antibodies (1:3,000, Cell Signaling, USA), the blot was detected using an imaging system (Amersham Pharmacia Biotech, Uppsala, Sweden).

### Statistical analysis

The experimental data were measured using GraphPad Prism 5 (GraphPad Software, Inc., La Jolla, CA, USA). Data were analyzed using one-way ANOVA followed by the Tukey test for comparisons among more than two groups of mice. All data were expressed as a means ± standard error of the mean. A *p*-value <0.05 was regarded as statistically significant.

## Results

### Investigation of association with EOM and allergic rhinitis through network construction

**Figure 1:**
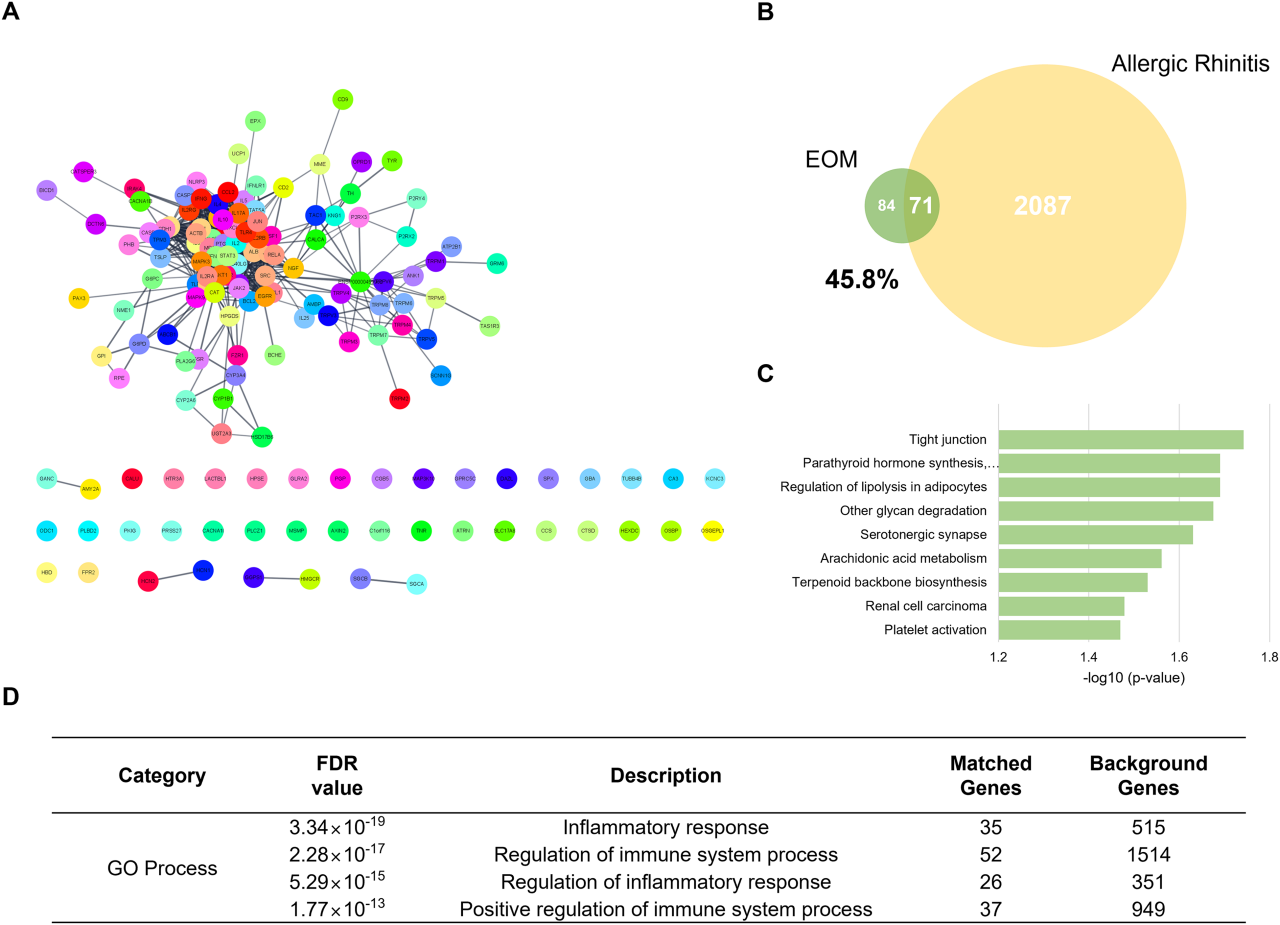
Network analysis of EOM. The PPI network of EOM Network. 150 nodes and 740 edges (**A**). The venn diagram of genes related to EOM and allergic rhinitis (**B**). Enrichment analysis of EOM network based on KEGG 2021 pathways, bar graph (**C**). Enrichment analysis of EOM network based on GO process (**D**). EOM, essential oils from the *Mentha piperita leaf*; KEGG, kyoto encyclopedia of genes and genomes; GO, gene ontology.

The EOM network constructing compounds menthone, menthol, and methyl acetate had 100, 100, and 14 co-efficient genes, respectively. After eliminating the duplicates, a total of 155 genes were yielded as chemical-gene co-occurrences of EOM. The EOM network was constructed with 155 nodes and 740 edges (Figure 1A). The target genes of the EOM were 155, and those of allergic rhinitis were 2087. The Venn diagram of EOM and allergic rhinitis targets showed that they had 71 identical targets in common. About 45.8% of the total target genes of EOM were matched to the target genes of allergic rhinitis (Figure 1B).

### Prediction of a significant pathway by enrichment analysis of KEGG and GO of target genes of EOM

The prediction of the significant pathway of EOM on allergic rhinitis was conducted by sorting the terms related to allergic rhinitis in enriched pathways derived from the EOM network. Among those pathways, “Tight junctions” in KEGG pathways were assigned to be a predicted related mechanism of the EOM network by false discovery rate (FDR) value <0.05 (Figure 1C). In addition, the GO process terms “Inflammatory response,” “Regulation of immune system process,” “Regulation of inflammatory response,” and “Positive regulation of immune system process” were shown as related responses and processes to the EOM network with an FDR value <0.05 (Figure 1D).

### Effects of EOM on histological changes in nasal septum tissues

**Figure 2:**
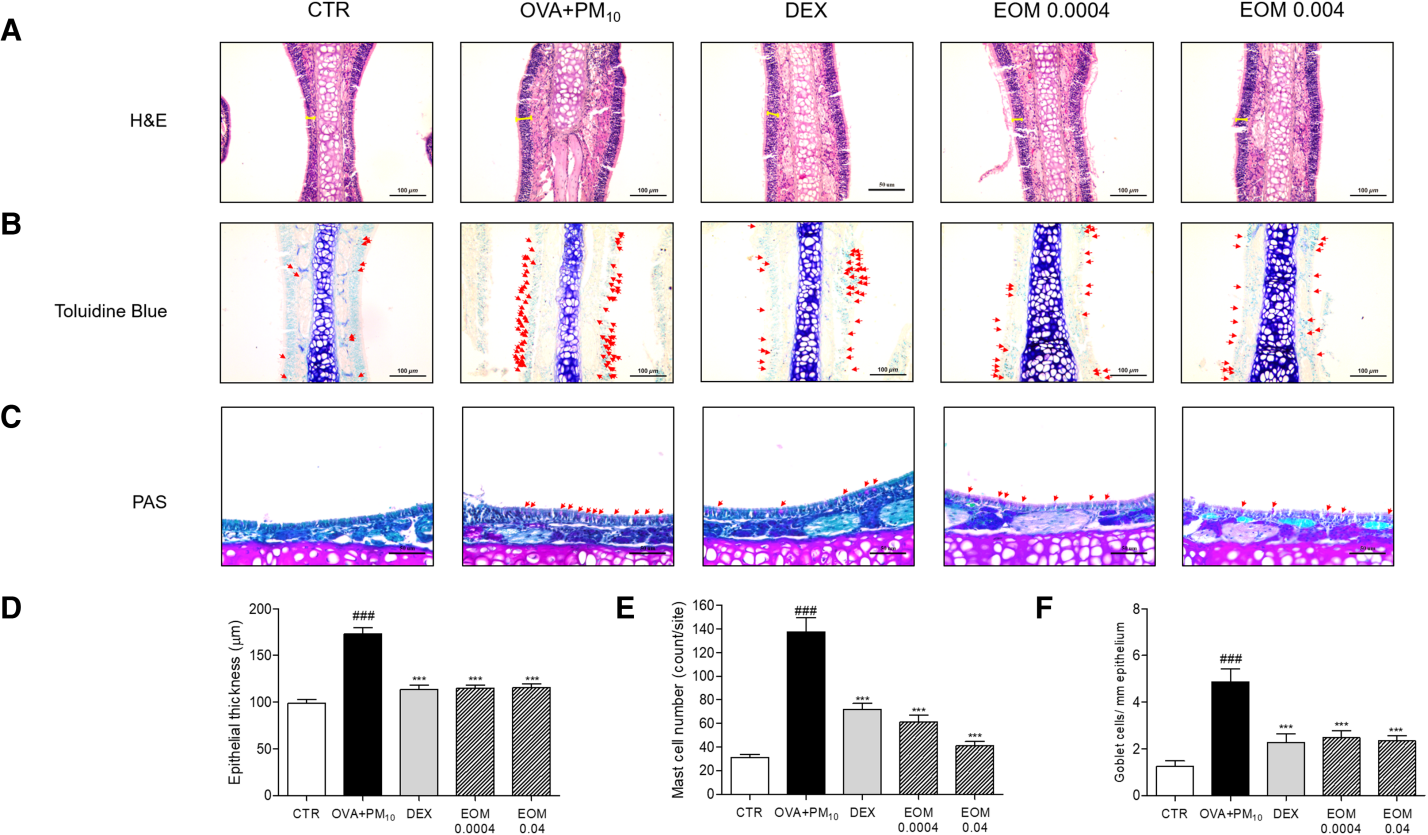
The effects of EOM on histopathological changes in nasal septum tissue during OVA and PM10-induced allergic rhinitis mice. The effects of EOM on histopathological changes in trachea tissue during OVA and PM10-induced allergic rhinitis mice. Trachea tissues were stained with hematoxylin and eosin (H&E) (**A**), toluidine blue (**B**), and periodic acid-Schiff (PAS) (**C**) in OVA and PM10-induced mice. Thickness of trachea epithelium (**D**), number of mast cell per site (**E**) and goblet cell number per area (**F**) were shown as relative quantified values. Results are presented as mean ± standard error of the mean. ^###^*p* < 0.001 vs. CTR group; *p < 0.05, **p < 0.01 and ***p < 0.001 vs. OVA and PM10-treated group. CTR, control; EOM, essential oils from the *Mentha piperita leaf*; OVA, ovalbumin.

We subsequently evaluated the effects of the EOM on the histological changes in the nasal septum using H&E, toluidine blue, and PAS staining. The thickening of the epithelial cells in the nasal septum tissues in the OVA + PM10 group was higher than that in the control group. There was a decrease of 33.44% and 33.21% in the epithelial thickness of EOM-treated mice (0.0004% v/v and 0.04% v/v) compared to the OVA + PM10-exposed mice (Figures 2A,D). As shown in Figures 2B,E, mast cell degranulation in the nasal septum tissues of the OVA + PM10 group was compared with the control group. In addition, a reduction of 55.37% in mast cells was observed in the EOM-treated group (0.0004% v/v and 0.04% v/v). Moreover, the results of the PAS staining demonstrated that the goblet cell secretion of the OVA + PM10 group was remarkably increased. We observed that increased goblet cells were markedly suppressed by EOM-treated groups (43.19% and 48.55%) (Figures 2C,F). The administration of DEX to the OVA + PM10 group significantly decreased the histological markers.

### Effects of EOM on nasal symptoms in a model of allergic rhinitis

**Figure 3:**
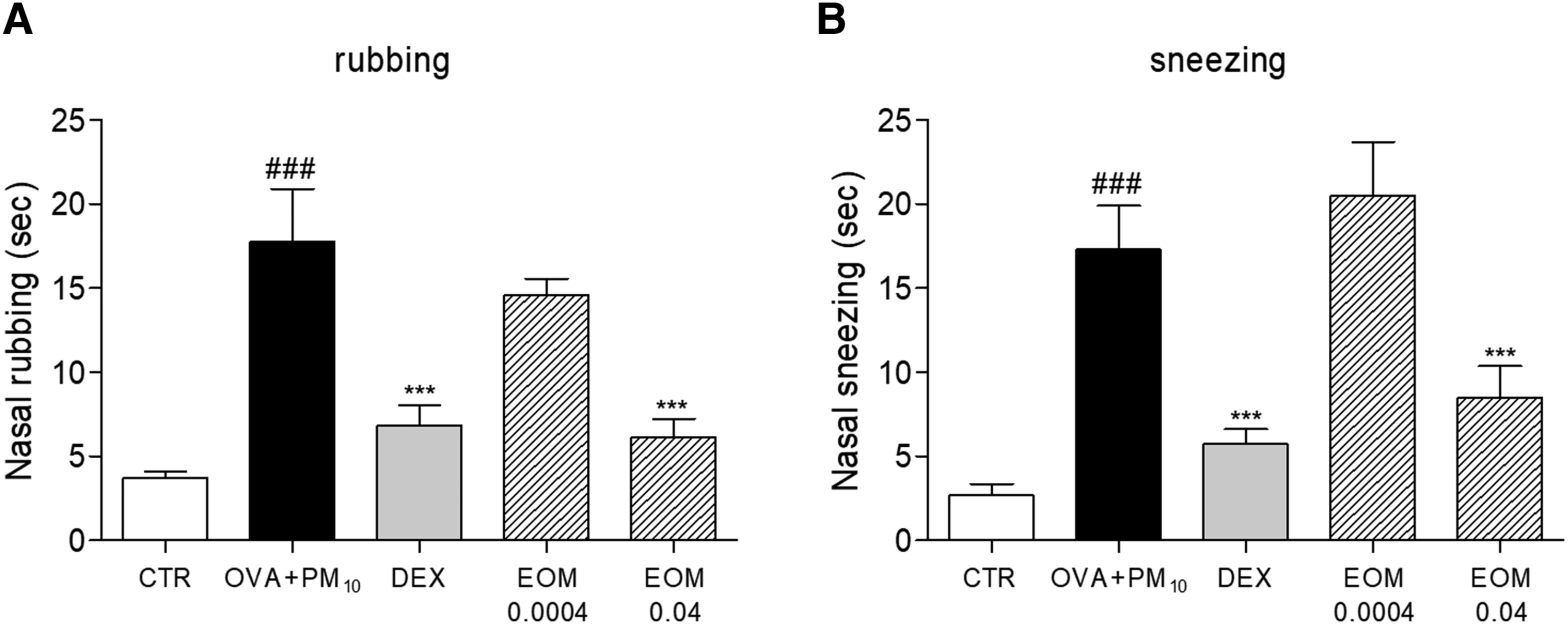
The effects of EOM on the nasal rubbing and sneezing symptom in OVA and PM10-induced model of allergic rhinitis (**A,B**). The nasal symptoms were evaluated after allergen challenge. EOM (0.0004, 0.04% v/v orally) was given to OVA and PM10-induced allergic rhinitis. Results are presented as mean ± standard error of the mean. ^###^*p* < 0.001 vs. CTR group; *p < 0.05, **p < 0.01 and ***p < 0.001 vs. OVA and PM10-treated group. CTR, control; EOM, essential oils from the *Mentha piperita leaf*; OVA, ovalbumin.

The occurrence of nasal rubbing and sneezing increased significantly in the allergic rhinitis models after intranasal administration. In Figure 3A, rubbing was decreased by 65.25% in the EOM 0.04 (% v/v) group compared to the control group. The DEX group used decreased nasal symptoms as a positive control. In addition, treatment in the EOM 0.04 (% v/v) group induced a reduction of 50.83% in the sneezing test (Figure 3B). This experiment also found no significant change in both EOM 0.0004 groups.

### Effects of EOM on the infiltration of inflammatory cells and IgE production in the NALF

**Figure 4:**
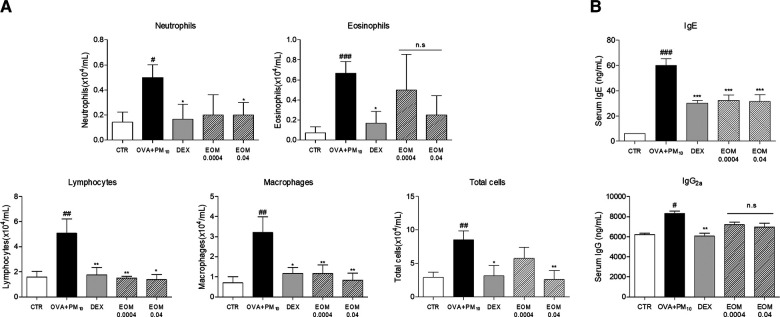
Effects of EOM on the infiltration of inflammatory cells and IgE production in the NALF. The inflammatory cells were counted by hemocytometer (**A**). EOM (0.0004, 0.04% v/v orally) was given to OVA and PM10-induced allergic rhinitis. The IgE and IgG2a were evaluated by ELISA (**B**). EOM (0.0004, 0.04% v/v orally) was given to OVA and PM10-induced allergic rhinitis. Results are presented as mean ± standard error of the mean. ^###^*p* < 0.001 vs. CTR group; *p < 0.05, **p < 0.01 and ***p < 0.001 vs. OVA and PM10-treated group. CTR, control; EOM, essential oils from the *Mentha piperita leaf*; OVA, ovalbumin; NALF, nasal lavage fluid; ELISA, enzyme-linked immunosorbent assay.

To demonstrate the role of EOM on nasal inflammation, the infiltration of cells in NALF was evaluated by ELISA. The total number of NALF cells in the OVA + PM10 group was reduced by the administration of EOM (0.0004% v/v and 0.04% v/v). The treatment with 0.04 (% v/v) of EOM significantly decreased the number of neutrophils. Moreover, approximately 70.4% and 72.8%, respectively, were lymphocytes in the OVA + PM10-induced group. Similar effects were demonstrated in the results of the macrophages and total cells (Figure 4A). In addition, the expression of IgE and IgG2a production was evaluated in the serum by ELISA. The level of IgE in the EOM-treated groups at concentrations of 0.0004 (% v/v) and 0.04 (% v/v) were reduced by 46% and 47.5%, respectively. However, IgG2a in the EOM-treated groups (0.0004% v/v, 0.04% v/v) was the same as in the OVA + PM10 group (Figure 4B).

### Effects of EOM on the expression of ZO-1 in nasal tissues

**Figure 5:**
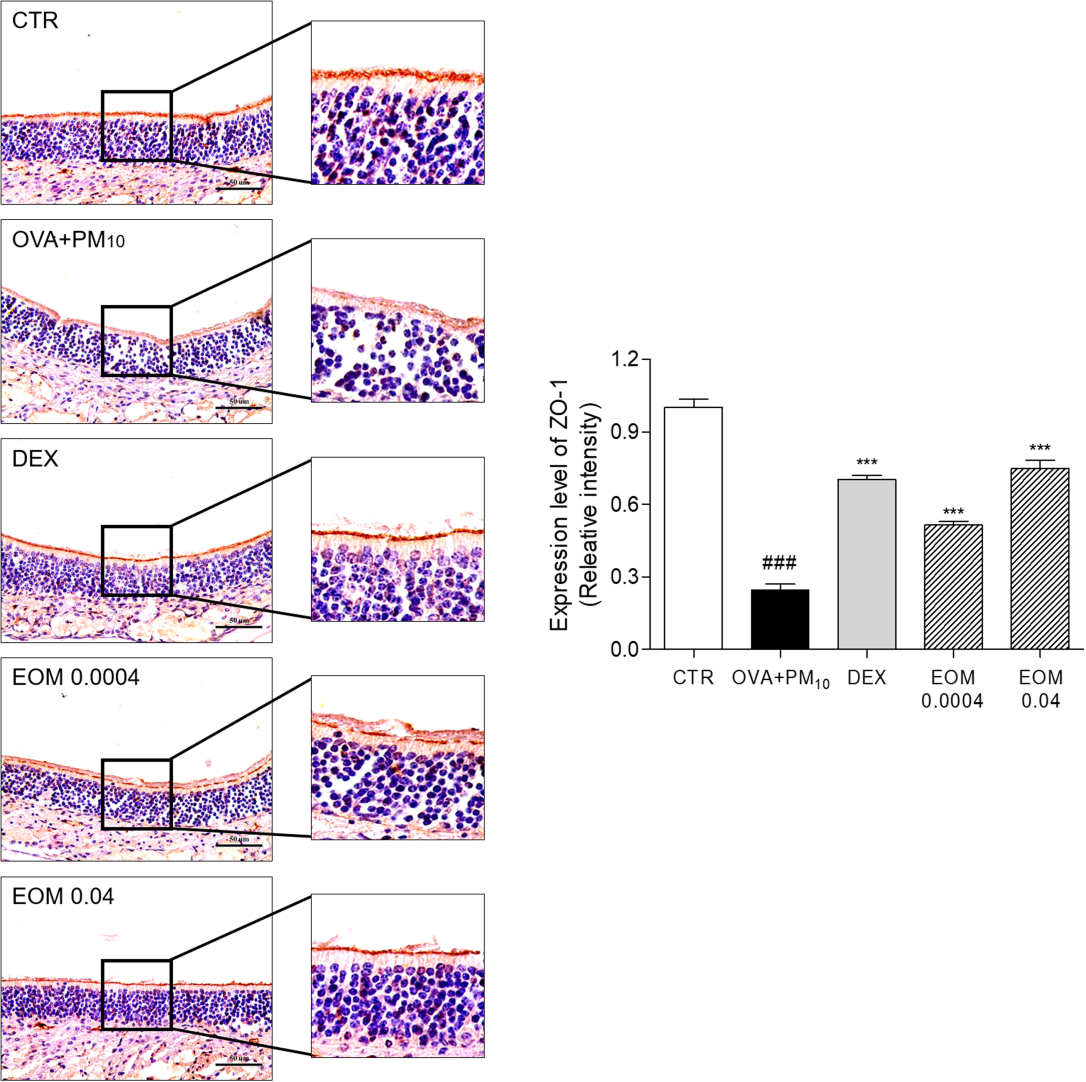
The effects of EOM on intracellular junctional proteins of nasal mucosa evaluated by immunohistochemistry. Nasal septum tissues were stained with IHC in OVA and PM10-induced mice. EOM (0.0004, 0.04% v/v orally) was given to OVA and PM10-induced allergic rhinitis. Results are presented as mean ± standard error of the mean. ^###^*p* < 0.001 vs. CTR group *p < 0.05, **p < 0.01 and ***p < 0.001 vs. OVA and PM10-treated group. CTR, control; EOM, essential oils from the *Mentha piperita leaf*; OVA, ovalbumin; IHC, immunohistochemistry.

IHC was performed to investigate the effect of EOM on regulating ZO-1. The expression of ZO-1 was reduced by 75.44% in the OVA + PM10-induced group. The administration of EOM 0.0004 and 0.04 (% v/v) improved the recuperation of the tight junctions by 2.08-fold and 3.04-fold. In addition, the treatment of DEX increased the expression of ZO-1 by 2.86-fold (Figure 5).

### Cytotoxicity of EOM on RPMI2650 nasal epithelial cells

**Figure 6:**
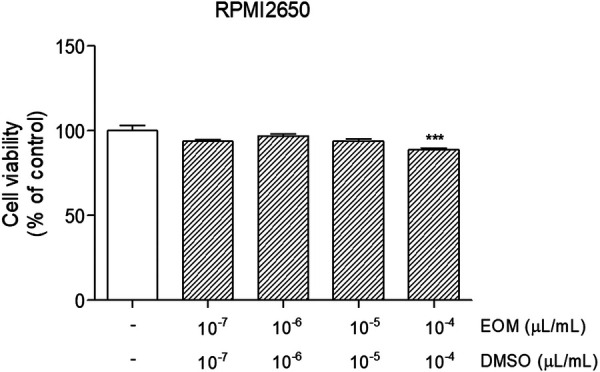
Cytotoxicity of EOM on RPMI2650 human nasal epithelial cells. Concentrations of EOM and the cell viability was determined using the MTT colorimetric assay. Results are presented as mean ± standard error of the mean ^###^*p* < 0.001 vs. CTR group *p < 0.05, **p < 0.01 and ***p < 0.001 vs. PM10-treated group. EOM, essential oils from the *Mentha piperita leaf*; MTT, 3-(4,5-dimethylthiazol-2-yl)-2,5-diphenyltetrazolium bromide, a tetrazole.

The effects of EOM on the viability of RPMI2650 cells were evaluated by MTT assay. As shown in Figure 6, cell viability did not decrease even in the EOM in the 10^−7^, 10^−6^, and 10^−5^/ml (% v/v)-treated group. These data revealed that EOM at specified concentrations was non-toxic to RPMI 2650 cells.

### Effects of EOM on the messenger RNA levels of tight junction-related factors in PM10-induced nasal epithelial cells

**Figure 7:**
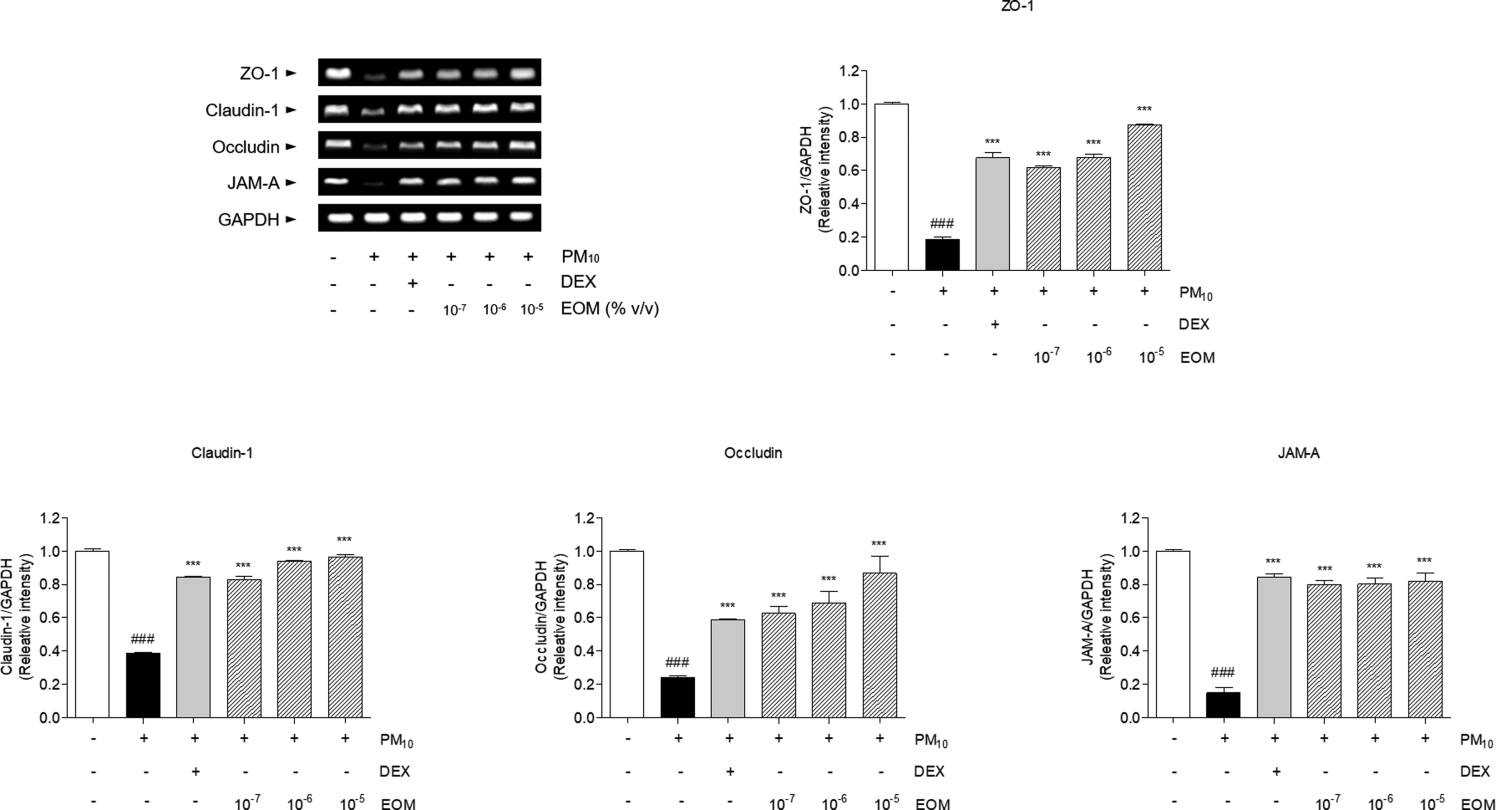
The expression of tight junction-related factors in PM10-treated RPMI2650 cells. The levels of tight junction related mRNA were visualized by RT-PCR analysis. Results are presented as mean ± standard error of the mean. ^###^*p* < 0.001 vs. CTR group *p < 0.05, **p < 0.01 and ***p < 0.001 vs. PM10-treated group. RT-PCR, reverse transcription polymerase chain reaction.

PM exposure to RPMI 2650 nasal epithelial cells induced the diminution of tight junction-related messenger RNA (mRNA) levels. Tight junction-related mRNA was decreased by stimulating PM10 in RPMI 2650 cells. The sensitization of PM10 significantly diminished the 74.74% level of ZO-1 in comparison with non-treated cells. The expression of ZO-1 in the 10^−7^, 10^−6^, and 10^−5^/ml (% v/v)-treated cells was significantly increased compared to the PM10-treated cells by 3.21-fold, 3.55-fold, and 4.51-fold. In addition, the expression of claudin-1 in the 10^−7^, 10^−6^, and 10^−5^/ml (% v/v)-treated cells was significantly increased compared to the PM10-treated cells by 2.15-fold, 2.44-fold, and 2.5-fold. The treatment of 10^−7^, 10^−6^, and 10^−5^/ml (% v/v) of EOM significantly increased the expression of occludin in the PM10-treated cells by 2.59, 2.85, and 3.59 times, respectively. The expression of JAM-A increased 5.3-fold, 5.32-fold, and 5.43-fold, respectively, compared with that of the negative control group. Further, the treatment of the DEX group markedly increased the levels of tight junction-related mRNA (Figure 7).

### Effects of EOM on the tight junction-related proteins in the PM10-induced nasal epithelial cells

**Figure 8:**
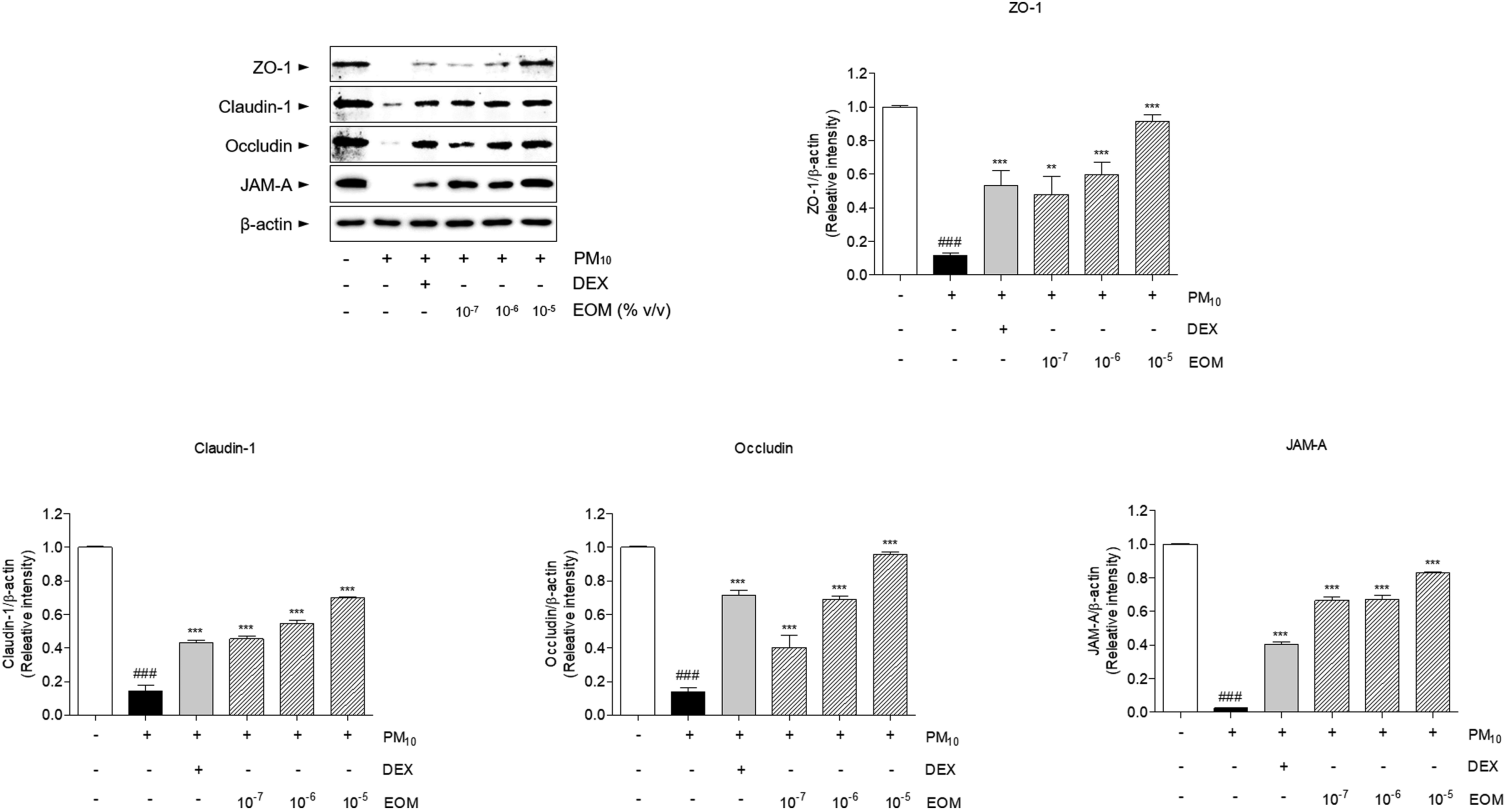
The expression of tight junction proteins in PM10-treated RPMI2650 cells. The levels of tight junction related protein were visualized by western blot analysis. Results are presented as mean ± standard error of the mean. ^###^*p* < 0.001 vs. CTR group *p < 0.05, **p < 0.01 and ***p < 0.001 vs. PM10-treated group.

The expression levels of ZO-1, claudin-1, occludin, and JAM-A in the RPMI 2650 cells treated with EOM were upregulated in a dose-dependent manner compared with the PM10-treated group. Tight junction-related proteins were decreased by stimulating PM10 in RPMI 2650 cells. The expression of ZO-1 in the 10^−7^, 10^−6^, and 10^−5^/ml (% v/v)-treated cells was significantly increased compared to the PM10-treated cells by 4.16-fold, 5.21-fold, and 7.96-fold, respectively. In addition, the expression of claudin-1 in the 10^−7^, 10^−6^, and 10^−5^/ml (% v/v)-treated cells was significantly increased compared to the PM10-treated cells by 3.15-fold, 3.8-fold, and 4.83-fold, respectively. The treatment of 10^−7^, 10^−6^, and 10^−5^/ml (% v/v) of EOM significantly increased the expression of occludin in PM10-treated cells by 2.96, 5.06, and 7.02 times, respectively. Expression of JAM-A increased 36.1-fold, 36.3-fold, and 42.8-fold, respectively, compared with that of the negative control group (Figure 8).

### Effects of EOM on the MAPK and Nf-kB-related proteins in the PM10-induced nasal epithelial cells

**Figure 9:**
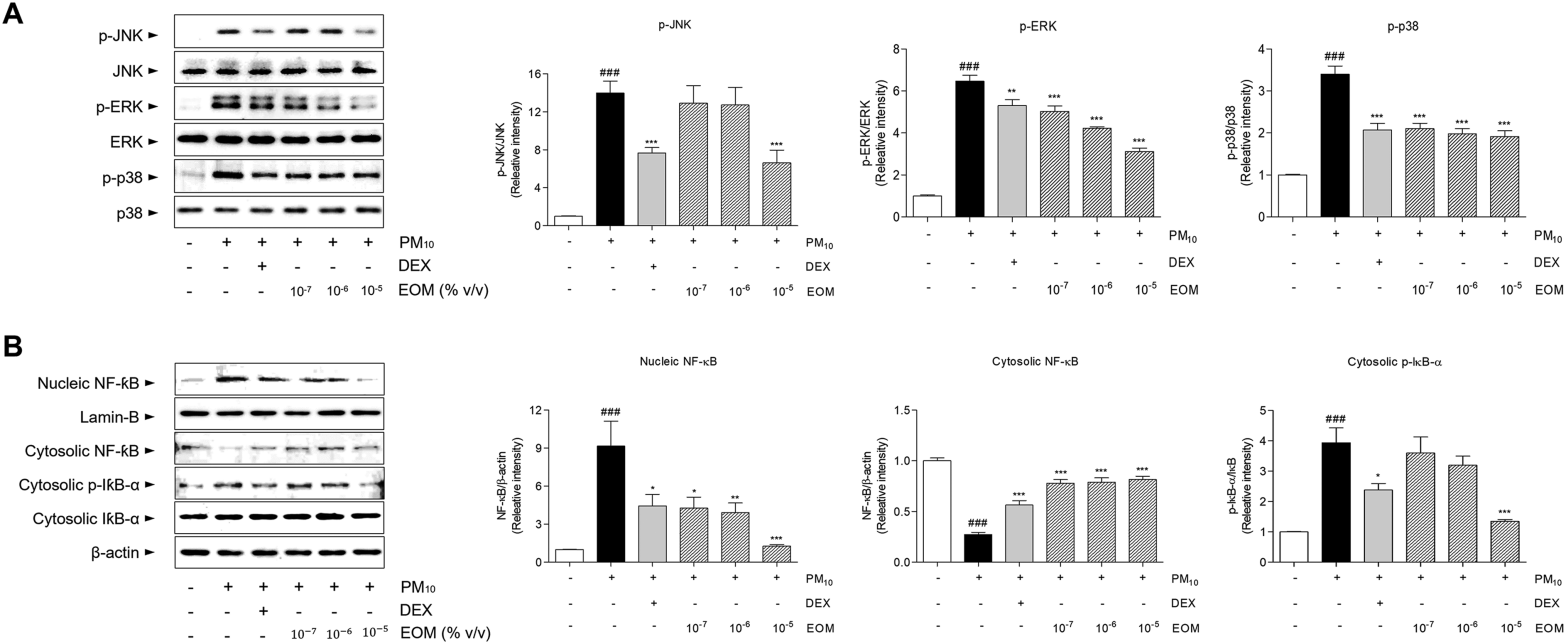
The expression of MAPK-NF-κB signaling in PM10-treated RPMI2650 cells. The expressions of phosphorylated MAPKs including JNK, ERK and p38 were shown with the quantified values (A). The expressions of nucleic and cytosolic NF-κB, and cytosolic IκB-α were shown with the quantified values (A). Results are presented as mean ± standard error of the mean. ###*p* < 0.001 vs. CTR group *p < 0.05, **p < 0.01 and ***p < 0.001 vs. PM10-treated group. MAPK, mitogen-activated protein kinase; NF-κB, nuclear factor kappa-light-chain-enhancer of activated B cell.

The exposure of EOM in the allergic rhinitis models regulated the mitogen-activated protein kinase (MAPK)-NF-κB signaling pathway. In addition, the exposure of DEX in the allergic rhinitis mice was evaluated in the MAPK-NF-κB signaling pathway. As shown in Figure 9A, the c-jun N-terminal kinase (JNK) and extracellular signal-regulated kinase (ERK) were phosphorylated by stimulating PM10 in RPMI 2650 cells. The phosphorylation of JNK in the 10^−7^, 10^−6^, and 10^−5^/ml (% v/v) of EOM-treated cells was significantly decreased by 7.59%, 9.01%, and 52.61%, respectively. The expression of ERK decreased by approximately 22.15%, 34.68%, and 51.78%, respectively, in the 10^−7^, 10^−6^, and 10^−5^/ml (% v/v) of EOM-treated cells. In addition, the treatment of all concentrations of EOM significantly reduced the phosphorylation levels of p38 (Figure 9A). Furthermore, the effects of the EOM-treated group on the NF-κB signaling pathway were observed by measuring the level of NF-κB in nucleic and cytosolic and the inhibitor of IκB-α in the cytosol by western blot. The treatment with EOM (10^−7^, 10^−6^, and 10^−5^/ml % v/v) group significantly regulated the NF-κB signaling pathway (Figure 9B).

## Discussion

Allergic rhinitis accompanies airway remodeling due to the disruption of tight junctions, and common inflammation features of airway remodeling include nasal obstruction, rhinorrhea, mucus secretion, and mast cell degranulation (21). Airway remodeling is defined as the modification of structural cells, thickness of epithelial tissue, and excessive mucus secretion of airway walls (22). It generally appears as hypersecretion of goblet cells and thickness of the basal membrane (23). This study speculates that allergic rhinitis might be linked to remodeling of the nasal structure. Through the results from histological changes, the present study showed that EOM treatment recovered nasal septum thickness and goblet cell hyperplasia in mice with OVA and PM-sensitized allergic rhinitis, suggesting that EOM has an inhibitory effect against nasal obstruction and rhinorrhea in allergic rhinitis. In addition, a common inflammatory response of allergic rhinitis is the degranulation of mast cells (24). An excessive increase the number of mast cells has been known to induce IgE synthesis and production of Th2-specific cytokine, IL-4, and IL-13 (25). Mast cells residing in the nasal region interact with the extracellular matrix proteins and adhesion molecules in nasal epithelial cells, thereby contributing to the late-phase allergic responses in allergic rhinitis (26). As shown in the histological changes, the number of mast cells was significantly reduced by EOM treatment in mice with OVA and PM-induced allergic rhinitis. From these findings, EOM inhalation effectively ameliorated the airway remodeling in allergic rhinitis.

Along with the airway remodeling, there are conjunctival symptoms including rhinorrhea, nasal itching, rubbing, sneezing, and nasal congestion in the development process of allergic rhinitis (27). Rubbing and sneezing are typical appearances of allergic diseases, especially in rhinitis, and those symptoms are mostly associated with allergen-specific IgE by airflow obstruction related to Th2-specific responses (28), while IgG2a is known to mainly be affected by the Th1 immune response in allergic diseases (29). In this study, EOM treatment inhibited allergic behaviors, rubbing, and sneezing in allergic rhinitis. In addition, EOM effectively inhibited the increase of serum IgE but not IgG2a. Those results demonstrated that EOM eased the allergic symptoms, including rubbing and sneezing, by inhibiting the production of IgE derived from Th2 immune responses. Moreover, it is well established that amelioration of inflammatory cell infiltration in NALF could interrupt the progression of allergic rhinitis. In particular, inflammatory cells including neutrophils, lymphocytes (30), and macrophages (31) are reported to be infiltrated in the development process of allergic rhinitis. In terms of eosinophil, it is known to be regulated by IL-5 and eotaxin rather than neutrophils, lymphocytes, and macrophages in allergic diseases (32). In this study, EOM significantly decreased the inflammatory cells, including neutrophils, lymphocytes, and macrophages but not eosinophils, in NALF, indicating that EOM was more effective in the inflammatory response through the disruption of the tight junctions than the inflammatory response caused by cytokines and eotaxin in allergic rhinitis.

In the process of airway remodeling in allergic rhinitis, the airway epithelial cells play a crucial role on inhaled environmental allergens and pathogens as structural barriers (33). A previous study reported the importance of the tight junctions on the development of intranasal drugs to enhance the bioavailability of “non-Lipinsky” small molecules, and peptide, protein, and oligonucleotide drugs (34). In addition, the tight junctions are known to be related to extracellular barrier permeability, which plays a crucial role in the maintenance of epithelial barrier function (35). Because this study considers that EOM might be associated with remodeling of the nasal structure due to the disruption of the structural barrier such as the tight junctions in allergic rhinitis, we used a network pharmacology analysis to predict the underlying mechanism of EOM on allergic rhinitis. The protein-protein interactions network (PPIN) obtained from menthone, menthol, and methyl acetate as the main components of EOM was constructed. According to the results of the network analysis, the 155 nodes and 740 edges were related to three main compounds in EOM. Further, the EOM network was expected to affect the tight junctions and inflammation through a functional enrichment analysis including the KEGG pathway and GO Process. From those results, we found that EOM is closely related to allergic rhinitis by regulating the tight junctions and inhibiting inflammatory responses. Following the predicted results using a network pharmacology, we then further investigated the molecular mechanism of EOM, especially focusing on tight junction regulation and inhibition of epithelial inflammation in vitro and in vivo.

Tight junctions in epithelial cells consist of primary constituents of transmembrane proteins, namely ZO-1, claudins, occludin, and JAMs (36). ZO-1, which directly interacts with intracellular tight junctions, including claudin-1, occludin, and JAM-A, is known to construct and regulate the structure of tight junctions (37). Claudin-1 is a defense protein that is essential for the regulation of paracellular permeability in the nasal barrier (38). Occludin is known to be maintained in the topmost layer of the pseudostratified columnar epithelium of the nasal mucosa to regulate permeability (39). In addition, JAM-A is essential for the regulation of the epithelial barrier in allergic rhinitis (40). EOM significantly recovered the expression levels of ZO-1 in the nasal tissue and nasal epithelial cells, demonstrating that the recovery of ZO-1 due to treatment with EOM helped to directly interact with intracellular tight junctions, including claudin-1, occludin, and JAM-A. Furthermore, mRNA and protein expression levels of claudin-1 and occludin were increased by EOM treatment in allergic rhinitis, indicating that EOM is effective in defending the nasal barrier. EOM improved the JAM-A that highly associated regulation of the nasal barrier in allergic rhinitis. In summary, the present study revealed that the EOM treatment recovered the expression of tight junctions, resulting in the recovery of the nasal epithelial barrier disruption that blocks the passage to allergens in allergic rhinitis.

To contribute to the tight junctions, one of the main inflammation pathways, the MAPK-NF-κB signaling pathway, has been reported to play major roles in allergic rhinitis (41). The activation of the MAPK signaling pathway, including ERK, JNK, and p38 phosphorylation, is mediated by claudin-1 and occludin (42), which have several phosphorylation sites on the carboxy-terminal tail, related to the regulation of MAPK (43). Downstream of MAPK, such as the NF-κB signaling pathway, is a major protein complex involved in the regulation of various immune response, including the inducement of allergic rhinitis (44). Based on those reports, inhibitors targeting MAPKs have been developed to reduce inflammation and the disruption of tight junctions (45). In this study, the effect of EOM on MAPK-NF-κB signaling pathway activation was investigated in PM10-stimulate human nasal epithelial cells. EOM treatment decreased phosphorylated ERK, JNK, and p38 in human nasal epithelial cells in the presence of PM10. The results of this study also showed that OVA and PM10-induced NF-κB activation was significantly regulated upon EOM treatment. These data showed that EOM treatment exerts an anti-allergic effect through the inactivation of the MAPK-NF-κB signaling pathway associated with tight junctions in allergic rhinitis. Thus, it can be suggested that the MAPK-NF-κB signaling pathway might participate in nasal epithelial barrier injury.

The key finding of this study is that, overall, the network analysis showed the predicted underlying mechanism of the potential effects of EOM as a therapeutic approach to treat allergic rhinitis to show the relationship between tight junction disruption and inflammation, and the efficacy of EOM on allergic rhinitis. DEX, which was used as a positive control in our study, is a steroid-based drug used to treat allergic rhinitis, exhibiting efficacies in the inhibition of an inflammatory mediator (46). There are numerous studies regarding the side effects of DEX, such as immune suppression, high blood pressure, and muscle weakness (47). Because essential oil inhalation therapy has long been safely used to treat various inflammatory diseases (48), we expected the inhalation of essential oil derived from a natural product using a nebulizer would be helpful to reduce the symptoms of allergic rhinitis instead of using steroid drugs. Inhalation of EOM reduced the disruption of the upper airway remodeling, such as nasal septum thickness, goblet cell hyperplasia, and infiltration of mast cells. EOM also recovered the symptoms of allergic rhinitis, especially the reduction of rubbing and sneezing. In addition, EOM effects the tight junctions and the epithelial barrier function, such as ZO-1, claudin-1, occludin, and JAM-A. In conclusion, it is suggested that EOM has advantages for the regulation of tight junction disruption of the nasal epithelial barrier and upper airway inflammation and in allergic rhinitis. Taken together, these findings might provide the therapeutic mechanism of EOM for the treatment of allergic rhinitis.

## Data Availability

The datasets presented in this study can be found in online repositories. The names of the repository/repositories and accession number(s) can be found below: https://pubchem.ncbi.nlm.nih.gov/, 1254; https://pubchem.ncbi.nlm.nih.gov/, 26447; https://pubchem.ncbi.nlm.nih.gov/, 27867.
