# Supplementary figures and images for: Protective effects of inhalation of essential oils from Mentha piperita leaf on tight junctions and inflammation in allergic rhinitis
Source: Front Allergy. 2022 Dec 12;3:1012183. doi: 10.3389/falgy.2022.1012183 (PMC9790934; doi:10.3389/falgy.2022.1012183)

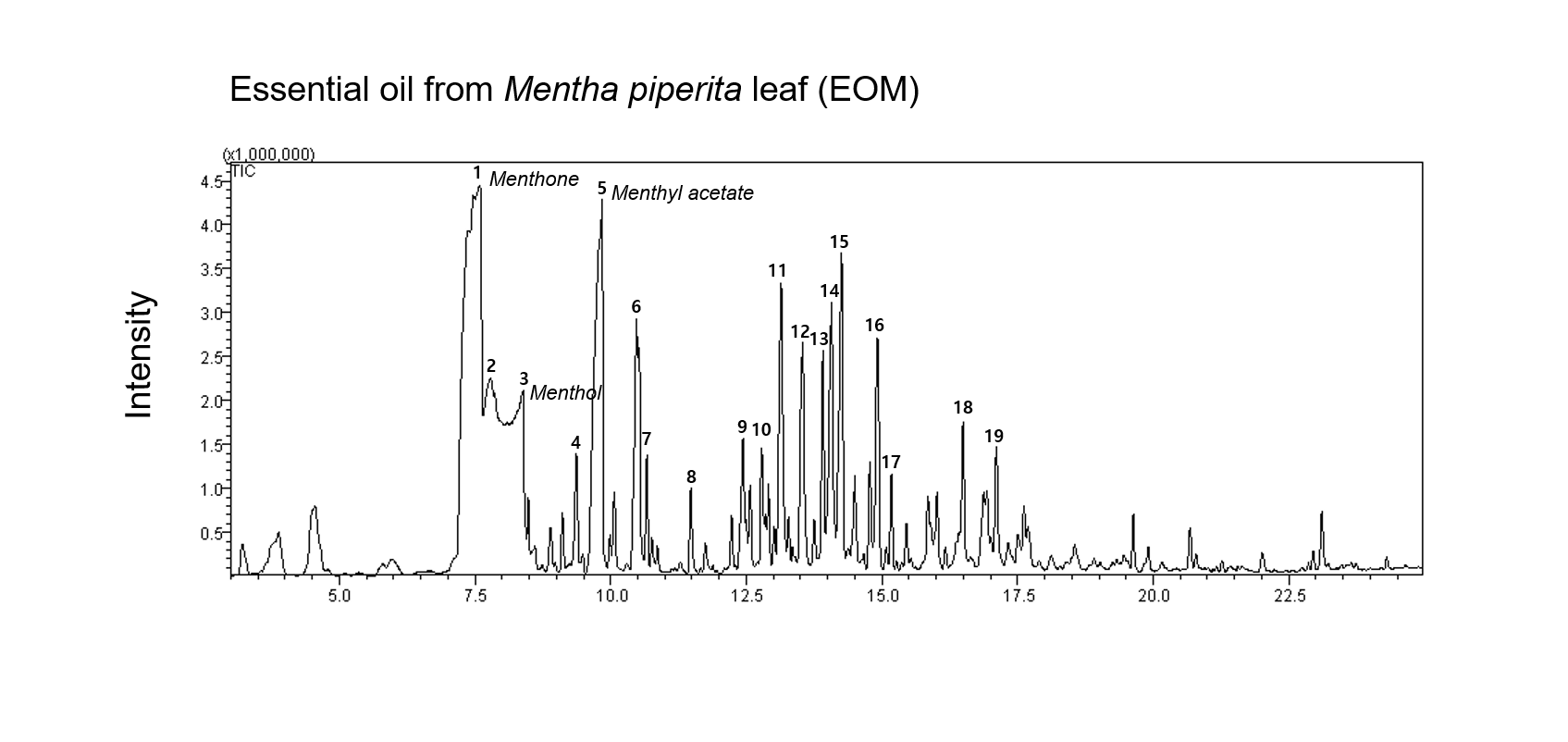

Supplement: Supplementary file 2 [file Image1.tif]
